# Researches on the Influence of Salt Added to the Food on the Development of Cattle

**Published:** 1848-05

**Authors:** 


*Researches on the influence of Salt added to the Food on the Developement of Cattle.* By M. Boussingault.—Some time since Boussingault published the commencement of some researches upon this subject, which tended to show that the influence of common salt, as an article of diet, is much less than is generally supposed. The experiments were made by selecting two lots of cattle and supplying them with a fixed quantity of food ; in the one lot with, and in the other without an addition of salt. The cattle were weighed previously to the commencement of the experiment, and again at its conclusion, and it was found that the gain in weight of the two lots, was scarcely appreciably different in amount. The author has extended these experiments in the present paper ; so that one of the lots of cattle has now been thirteen months deprived of salt, and his former results are fully confirmed. He has found that the consumption of 100 kilogrammes of hay, caused an increase of 7.19 kilogrammes of weight on those which were supplied with salt ; and of 6.83 in those which got none. This difference is extremely trifling, and it is probable that it would be as great, or even greater, between different lots fed in precisely the same manner ; and in an economic point of view, M. Boussingault observes, that the trifling gain in the former lot would not cover the expense of the salt with which they were supplied.

It is remarkable, however, that though the absence of salt excited no influence upon the growth of the cattle, it had a well-marked effect upon their general appearance. In those which were deprived of salt, the coat become rough, and the hair even detached itself in some places, and they were dull and inactive ; while those of the other lot presented a fine smooth coat, and were perfectly lively and active, and, from their more favourable appearance, would unquestionably have brought a higher price in the market.—*London Monthly Journal from Comptes Rendus.*

